# Diagnostic Accuracy of Circular RNAs in Different Types of Samples for Detecting Hepatocellular Carcinoma: A Meta-Analysis

**DOI:** 10.3389/fgene.2021.794105

**Published:** 2021-12-21

**Authors:** Guilin Nie, Dingzhong Peng, Bei Li, Jiong Lu, Xianze Xiong

**Affiliations:** Department of Biliary Surgery, West China Hospital of Sichuan University, Chengdu, China

**Keywords:** circular RNAs, exosome, serum/plasma, hepatocellular carcinoma, diagnosis, meta-analysis

## Abstract

The lack of accurate biomarkers impeded the screening, diagnosis and early treatment of hepatocellular carcinoma (HCC). As a result of the development of high-throughput transcriptome analysis techniques, circular RNAs, a newly discovered class of noncoding RNAs, were recognized as potential novel biomarkers. This meta-analysis was performed to update the diagnostic roles of circular RNAs for HCC. We acquired 23 articles from PubMed, Web of Science, EMBASE, and Cochrane Library databases up to September 2021. The overall sensitivity was 0.80 (95% CI: 0.77–0.84), and the specificity was 0.83 (95% CI: 0.79–0.85), with an AUC of 0.88 (0.85–0.91). Considering of the significant heterogeneity, studies were divided into four groups based on the control types. The circular RNAs in exosomes had a sensitivity of 0.69 (95% CI: 0.61–0.75), and a highest specificity of 0.91 (95% CI: 0.83–0.96). The pooled sensitivity of circular RNAs in serum/plasma was 0.84 (95% CI: 0.81–0.87), and the pooled specificity was 0.83 (95% CI: 0.79–0.86). The pooled sensitivity of circular RNAs distinguishing tumor tissue from chronic hepatitis/cirrhosis tissues was 0.56 (95% CI: 0.48–0.64), and specificity was 0.76 (95% CI: 0.67–0.82). When the controls were adjacent tissues, the sensitivity was 0.78 (95% CI: 0.70–0.84), and the specificity was 0.78 (95% CI: 0.71–0.85). Hsa_circ_0001445 with a pooled sensitivity of 0.81, a specificity of 0.76 and an AUC of 0.85 in two studies, might be a suitable diagnostic blood biomarker for HCC. Relying on function in HCC, the AUC of subgroups were 0.88 (95%CI: 0.84–0.90) (function group) and 0.87 (95%CI: 0.84–0.90) (unknown function group). As for only reported in HCC or not, these circular RNAs had an AUC of 0.89 (95%CI: 0.86–0.91) (only in HCC) and 0.85 (95%CI: 0.82–0.88) (not only in HCC). In conclusion, the results suggested that circular RNAs were acceptable biomarkers for detecting HCC, especially those circular RNAs existing in exosomes or serum/plasma.

## 1 Introduction

Hepatocellular carcinoma (HCC) is the fourth leading cause of cancer-related death worldwide. In 2020, 905,677 new cases and 830,180 deaths from HCC were reported, which accounted for 75–85% of cases of primary liver cancer (Sung et al., 2021). Furthermore, it is the fourth most common malignancy and the second leading cause of tumor-related death in China (Chen et al., 2016). According to the recommendation of guidelines (European Association for the Study of the Liver, 2018; Marrero et al., 2018), HCC will be treated more efficaciously if it is diagnosed early. Aside from alpha-fetoprotein (AFP), a biomarker whose sensitivity and specificity for detecting HCC ranges from 39 to 64% and 76–91% (Oka et al., 1994; Okuda et al., 2000; Marrero and Lok, 2004), and complex ultrasound, respectively, a class of more sensitive and specific diagnostic markers of HCC are urged to improve the accuracy of early diagnosis.

Endogenous circular RNAs are characterized by covalently closed-loop noncoding RNAs (Jeck et al., 2013), giving the circular RNAs higher stability than their linear counterparts, which prevents exonuclease-mediated degradation (Jeck et al., 2013). Circular RNAs are expressed widely in all human tissues, but their functions may be specific to cells and tissues (Salzman et al., 2013). Since the recent discovery of circular RNAs, interest in their clinical characteristics and pathologic mechanisms (especially in solid tumors) has increased. Despite their widespread existence, only a minor fraction of circular RNAs possessing biological functions has been identified. Nevertheless, this limitation cannot prevent circular RNAs from becoming efficacious therapeutic targets for tumors. Most circular RNAs reportedly act as microRNA (miRNA) “sponges” (Hansen et al., 2013), whereas others interact with proteins to regulate the function of proteins (Li Z. et al., 2015), change the stability of mRNAs (Chen et al., 2019), or to code proteins (Zhang M. et al., 2018).

Moreover, the stability and wide existence of circular RNAs in tissues and blood suggest that they may be effective diagnostic biomarkers of HCC (Shen et al., 2021). Recent studies have found their existence not only in tumor and blood, but also in exosomes, saliva, urine and bile (Li Y. et al., 2015; Bahn et al., 2015; Kölling et al., 2019; Wen et al., 2020; Xu et al., 2021), making them ideal noninvasive biopsy biomarker candidates. Exosome-derived circular RNAs (Wang et al., 2019) or multi-circular RNAs diagnostic models (Yu et al., 2020) have been created to improve the diagnostic accuracy of circular RNAs. Also, a sensitive, selective, and stable integrated electrochemical point-of-care (POCT) platform for detection of circular RNAs has been invented to achieve the rapid screening and detection of HCC (Zhang et al., 2021). The development and application of new detection technologies of circular RNAs provides a bright future in circular RNAs field. We undertook a meta-analysis to summarize the latest advances of diagnostic values and characteristics of circular RNAs in HCC and process a deeper exploration centered on the roles of circular RNAs in blood and exosomes, and different diagnostic values from different tissues (cirrhosis, hepatitis, and adjacent tumor tissues). Meanwhile, the problem if it would be different in diagnostic accuracy of functional circular RNAs or not and special-expression circular RNAs in HCC or not were involved in our analysis.

## 2 Methods

### 2.1 Search Strategy

Two investigators (Dingzhong Peng and Bei Li) searched relevant studies in PubMed, Web of Science, Cochrane Library, and EMBASE up to September 2, 2021, following the Preferred Reporting Items for Systematic reviews and Meta-Analyses (PRISMA) (Page et al., 2021) protocols. We used the following keywords: “circRNA or circular RNA” and “liver cancer or liver carcinoma or hepatocellular carcinoma or HCC”. We also searched the reference lists of selected articles or contacted the authors to obtain more details.

### 2.2 Criteria for Study Selection

The inclusion criteria were: 1) the study explored the relationship between circular RNAs and HCC; 2) the diagnosis of HCC was made on the basis of histopathology; 3) detection method for ncRNA profiling was clearly defined in the article; 4) case–control study or cohort study; 5) the study contained adequate information so that the prevalence of true-positives (TPs), true-negatives (TNs), false-positives (FPs), and false-negatives (FNs) could be calculated for the diagnosis.

The exclusion criteria were: 1) letters, case reports, meta-analysis, review articles, or animal studies; 2) studies not relevant to circular RNAs or HCC; 3) unavailable/incomplete data or missing researchers; 4) the studies written in a language other than English.

### 2.3 Extraction and Quality Assessment of Data

Two investigators extracted relevant data for analyses independently. The information extracted was: 1) first author; 2) publication year; 3) type of cancer and circular RNAs; 4) type and size of specimen; 5) assay method for circular RNAs; 6) cutoff value of circular RNAs; 7) study location; 8) TP, TN, FP, FN, and AUC for diagnostic analyses; 9) circular RNAs expression. The quality of included diagnostic studies was evaluated using Quality Assessment of Diagnostic Accuracy Studies 2 (QUADAS-2) (Whiting et al., 2011) and showed in Supplementary Figure S1. A third investigator participated in the process if problems arose.

### 2.4 Statistical Analysis

Data were analyzed using the “midas” plugin of STATA 15.0 (STATA, College Station, TX, United States ) and Review Manager 5.4 (https://training.cochrane.org/online-learning/core-software-cochrane-reviews/revman/). The pooled sensitivity and specificity (with 95% confidence intervals (CIs)) were calculated to determine the diagnostic value of parameters. The threshold effect between included studies was assessed by the correlation coefficient and *p*-value. *p* < 0.05 indicated the existence of a threshold effect. The hierarchical summary receiver operating characteristics (HSROC) model was used to ameliorate the threshold effect and plot SROC in midas (Rutter and Gatsonis, 2001). Heterogeneity was estimated using Cochran’s Q test and I^2^ (Higgins et al., 2003). A random-effects model was adopted if I^2^ >50% or *p* < 0.10. The pooled diagnostic odds ratio (DOR), positive likelihood ratio (+LR), and negative likelihood ratio (−LR) were calculated for deeper analyses of heterogeneity. Then, subgroup analyses were conducted if high heterogeneity was present. We used Fagan’s nomogram plot analysis to evaluate the changes between pre-test probability and post-test probability (the probability of having the disease after the result of the index test had been obtained). A potential publication bias of articles related to HCC diagnosis was evaluated using Deeks’ funnel-plot asymmetry test. *p* < 0.10 indicated significant asymmetry and a publication bias.

## 3 Results

### 3.1 Included Studies

**FIGURE 1:**
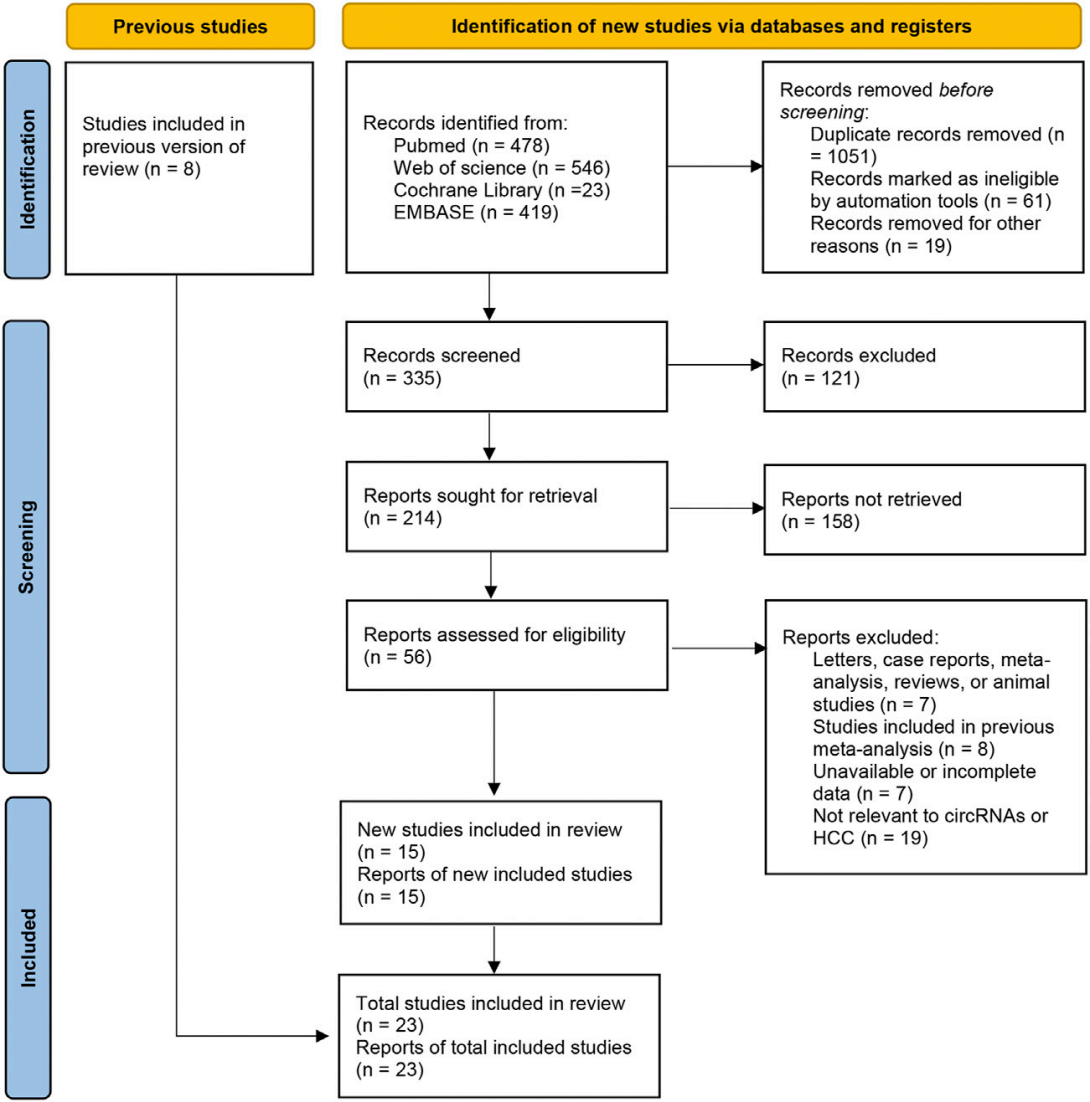
Flow-process diagram of the study selection process.

Twenty-three studies were included in our meta-analysis (Qin et al., 2016; Shang et al., 2016; Yao et al., 2017; Zhang et al., 2018b; Chen et al., 2018; Zhang et al., 2018c; Fu et al., 2018; Matboli et al., 2018; Yao et al., 2018; Li Z. et al., 2019; Jiang et al., 2019; Qiao et al., 2019; Sun S. et al., 2020; Sun X.-H. et al., 2020; Chen et al., 2020; Gao et al., 2020; Wei et al., 2020; Wu et al., 2020; Yu et al., 2020; Zhu et al., 2020; Guo et al., 2021; Liu et al., 2021; Wang et al., 2021). The types of clinical samples were tumor tissue, adjacent tissue, tissue from cirrhosis patients, tissue from hepatitis patients, serum/plasma, and exosome. All studies focused on circular RNAs and measured the levels of circular RNAs by real time reverse-transcription-quantitative polymerase chain reaction. The number of samples ranged from 72 to 278. Sufficient data were selected from the articles for calculation of pooled sensitivity, specificity, and other statistical indicators. The details and process are shown in Supplementary Table S1 and Figure 1.

### 3.2 Primary meta-Analysis

**FIGURE 2:**
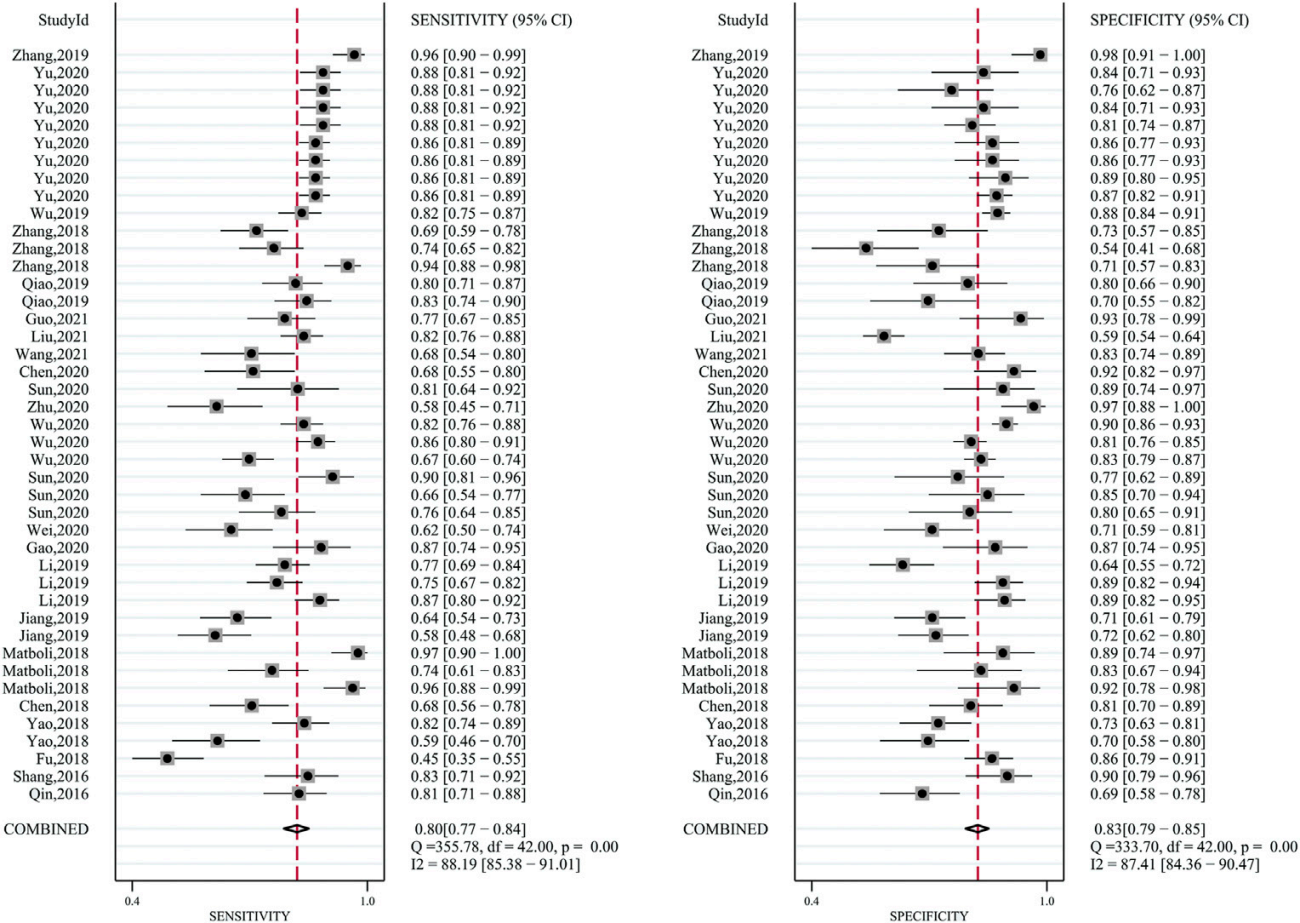
Forest plots for diagnostic accuracy of circular RNAs in hepatocellular carcinoma (HCC).

**FIGURE 3:**
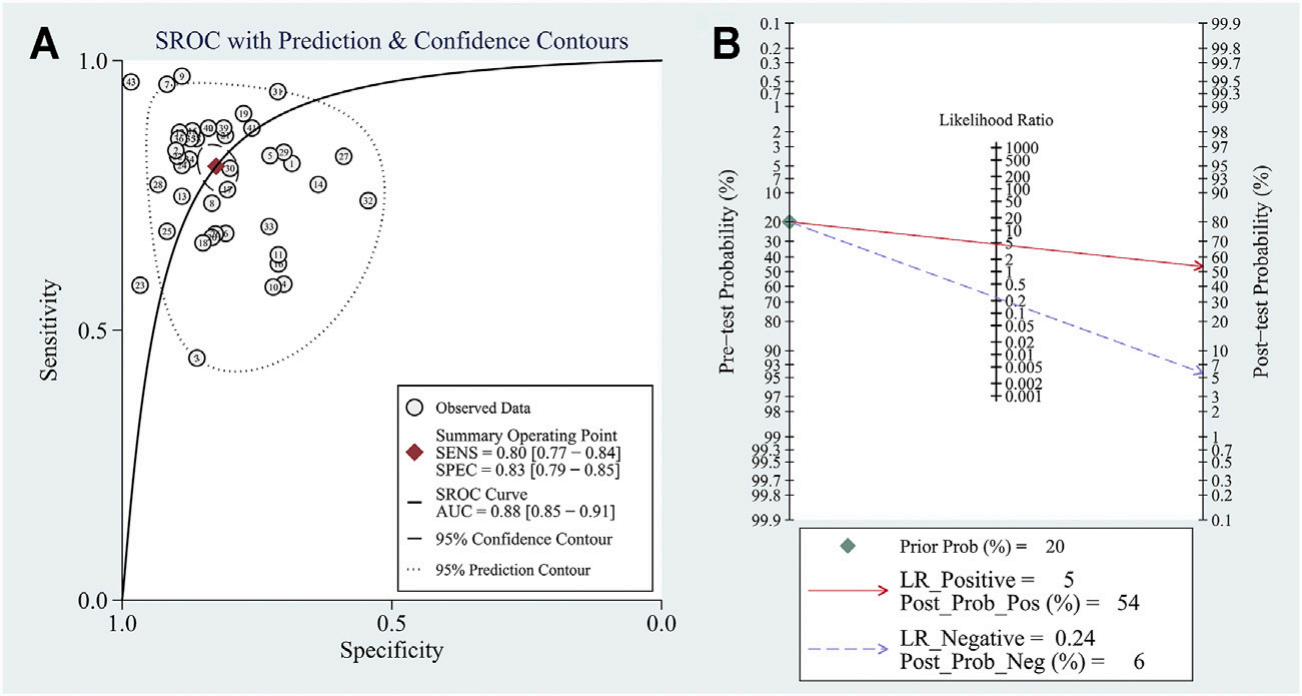
**(A)** The summary receiver operator characteristic (SROC) for circular RNAs in hepatocellular carcinoma; **(B)** Fagan plot analysis to evaluate the clinical utility of circular RNAs.

The pooled sensitivity was 0.80 (95%CI: 0.77–0.84). The pooled specificity was 0.83 (95%CI: 0.79–0.85) (Figure 2). The pooled DOR, +LR, and −LR was 19 (95%CI: 14–27), 4.6 (95%CI: 3.8–5.5), and 0.24 (95%CI: 0.20–0.29), respectively (Table 1). The correlation coefficient of the threshold effect of circular RNAs was 0.28 (*p* < 0.08): a threshold effect was absent. The pooled AUC arising from the HSROC model was 0.88 (0.85–0.91). Cochran’s Q was 125.75 (*p* < 0.001), and I^2^ was 98%, which showed considerable heterogeneity (Figure 3A). Thus, a subgroup analysis for the types of samples was processed. With a pre-test probability of a positive circular RNAs of 20%, the post-test probability of positive circular RNAs giving positive and negative results was 54 and 6%, respectively (Figure 3B).

**TABLE 1:** Subgroup analysis of diagnostic accuracy of circular RNAs in HCC.

	Sen	Spe	PLR	NLR	DOR	AUC	heterogeneity	
	95%CI	95%CI	95%CI	95%CI	95%CI	95%CI	I^2^	P
Type of sample								
Exosomes	0.69 (0.61,0.75)	0.91 (0.83,0.96)	7.8 (4.0,15.2)	0.34 (0.28,0.43)	23 (11,47)	0.82 (0.78,0.85)	32%	0.115
Serum/plasma	0.84 (0.81,0.87)	0.83 (0.79,0.86)	4.9 (4.0,6.1)	0.19 (0.16,0.23)	26 (18,38)	0.90 (0.87,0.93)	97%	< 0.001
HCC vs adjacent tissues	0.79 (0.72,0.85)	0.79 (0.72,0.84)	3.7 (2.7,5.0)	0.27 (0.19,0.37)	14 (8,25)	0.86 (0.82,0.88)	21%	0.181
HCC vs hepatitis tissues	0.56 (0.49,0.64)	0.76 (0.67,0.82)	2.1 (1.8,2.6)	0.58 (0.50,0.66)	4 (3,6)	0.70 (0.66,0.74)	80%	0.004
Function								
Yes	0.82 (0.77,0.86)	0.80 (0.73,0.86)	4.1 (2.9,5.8)	0.23 (0.18,0.30)	18 (10,31)	0.88 (0.84,0.90)	95%	< 0.001
No	0.75 (0.67,0.82)	0.84 (0.80,0.87)	4.6 (3.5,6.0)	0.30 (0.21,0.41)	15 (9,27)	0.87 (0.84,0.90)	95%	< 0.001
Only in HCC								
Yes	0.79 (0.71,0.84)	0.84 (0.80,0.88)	4.9 (3.7,6.7)	0.25 (0.18,0.35)	19 (11,35)	0.89 (0.86,0.91)	98%	< 0.001
No	0.79 (0.73,0.83)	0.78 (0.70,0.85)	3.6 (2.6,5.0)<	0.27 (0.21,0.35)	13 (8,21)	0.85 (0.82,0.88)	95%	< 0.001
Overall	0.80 (0.79,0.85)	0.83 (0.79,0.85)	4.6 (3.8,5.5)	0.24 (0.20,0.29)	19 (14,27)	0.88 (0.85,0.91)	98%	< 0.001

Abbreviations: Sen: sensitivity; Spe: specificity; +LR: positive likelihood ratio; -LR: negative likelihood ratio; DOR: diagnostic OR; AUC: area under the curve; HCC: hepatocellular carcinoma.

### 3.3 Subgroup Analysis

The significant heterogeneity that we encountered prompted us to undertake subgroup analyses. Four groups were created: sample type of exosomes; serum/plasma; HCC tissue *vs* tissue from cirrhosis cases or tissue from cases with chronic hepatitis; HCC tissue *vs* adjacent tissue. The sensitivity, specificity, AUC, and other statistical indicators were calculated and compared (Table 1). Besides that, we also divided all of them into other kind of subgroups, based on function in HCC or not and only studied in HCC or not. The same analysis methods were possessed.

#### 3.3.1 Types of Samples

##### 3.3.1.1 Exosomes

**FIGURE 4:**
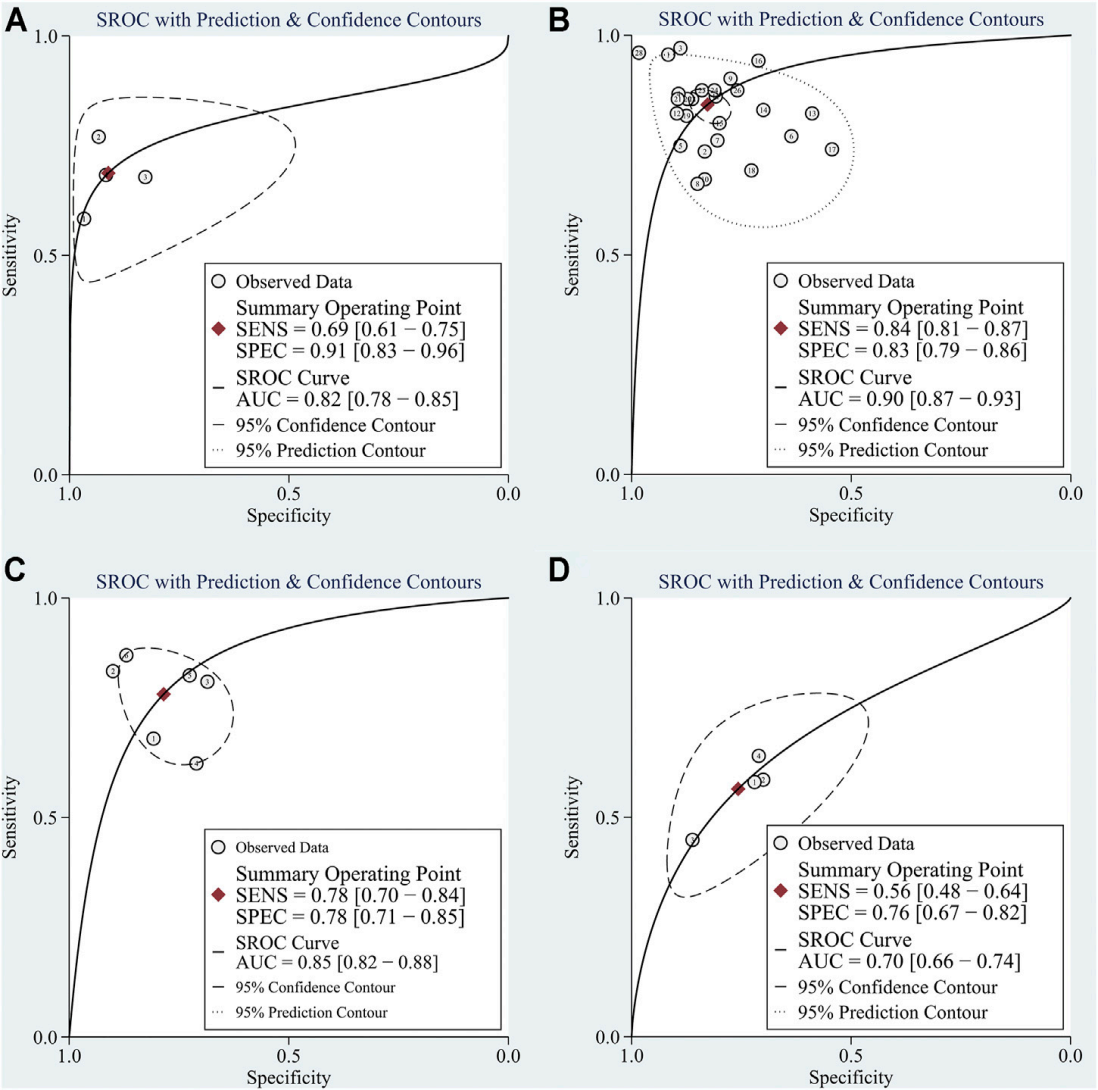
The summary receiver operator characteristic (SROC) for circular RNAs in **(A)** exosomes; **(B)** serum/plasma; **(C)** HCC tissue vs adjacent tissue; **(D)** HCC tissues vs tissues from cirrhosis or chronic hepatitis cases.

Four studies focused on the diagnostic value of exosome-derived circular RNAs. All of those studies were published in 2020–2021. The pooled sensitivity was 0.69 (95%CI: 0.61–0.75). The pooled specificity was 0.91 (95%CI: 0.83–0.96). The pooled DOR, +LR, and −LR was 23 (95%CI: 11–47), 7.8 (95%CI: 4.0–15.2), and 0.34 (95%CI: 0.28–0.43), respectively. Fagan’s nomogram plot analysis revealed values of 66% (positive) and 8% (negative) (Supplementary Figure S2A). The correlation coefficient of the threshold effect was −0.73 (*p* = 0.53): the threshold effect was absent. The pooled AUC was 0.82 (0.78–0.85) (Figure 4A). Cochran’s Q was 2.942 (*p* = 0.115) and I^2^ was 32%, which indicated low heterogeneity.

##### 3.3.1.2 Serum/Plasma

**FIGURE 5:**
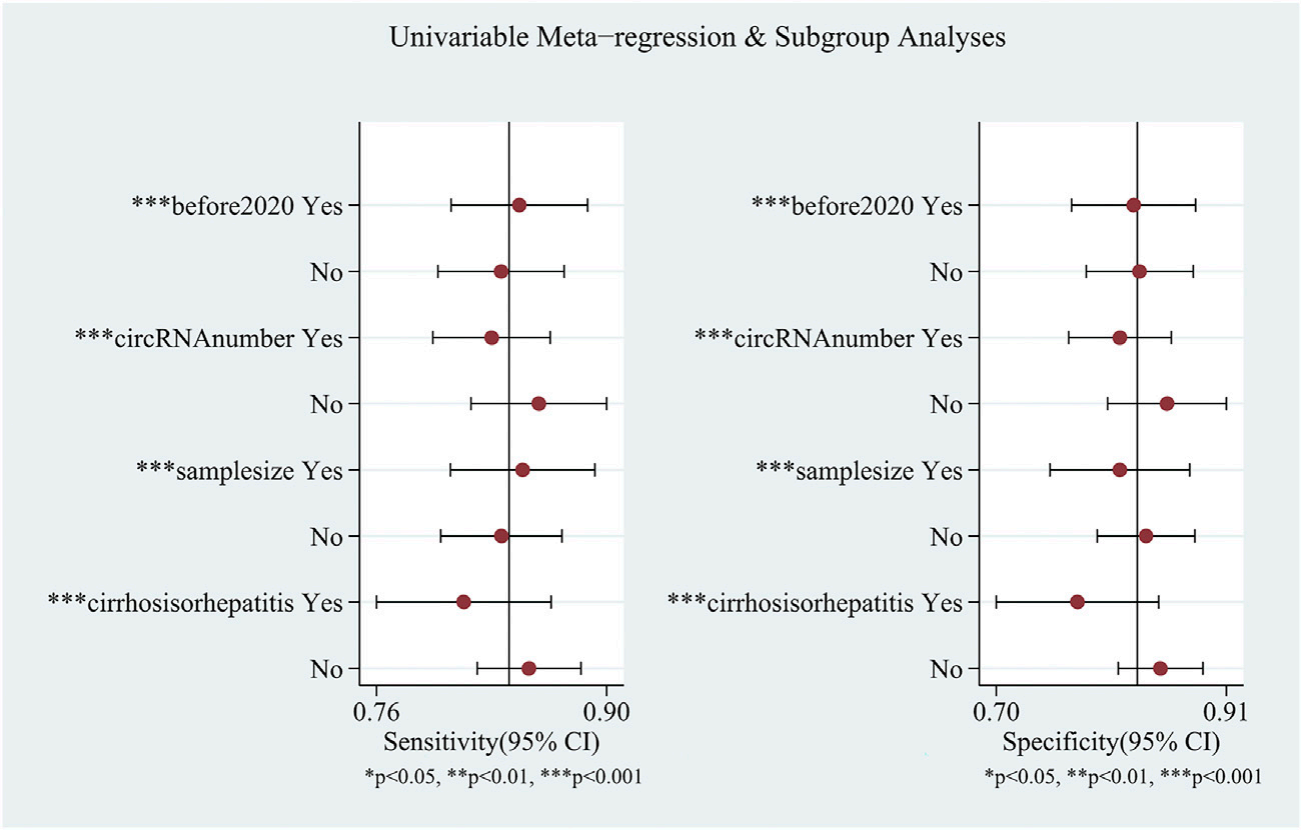
Meta-regression for the heterogeneity existing in circular RNAs in plasma/serum.

Ten studies explored the diagnostic accuracy of circular RNAs in serum/plasma. Most of these studies were published in 2020–2021. The pooled sensitivity was 0.84 (95%CI: 0.81–0.87). The pooled specificity was 0.83 (95%CI: 0.79–0.86). The pooled DOR, +LR, and −LR was 26 (95%CI: 18–38), 4.9 (95%CI: 4.0–6.1), and 0.19 (95%CI: 0.16–0.23), respectively. Fagan’s nomogram plot analysis revealed values of 55% (positive) and 5% (negative) (Supplementary Figure S2B). The correlation coefficient of the threshold effect was 0.44 (*p* = 0.20): the threshold effect was considered absent. The pooled AUC was 0.90 (0.87–0.93) (Figure 4B). Cochran’s Q was 59.459 (*p* < 0.001), and I^2^ was 97%, which indicated high heterogeneity. To solve the heterogeneity, we processed a meta-regression based on publication year (before 2020 or not), circular RNAs numbers (single or multiple), high risk patients (with cirrhosis or hepatitis or not), and sample size (< 200 or ≥200). We found that all of these could influence the heterogeneity (Figure 5).

**FIGURE 6:**
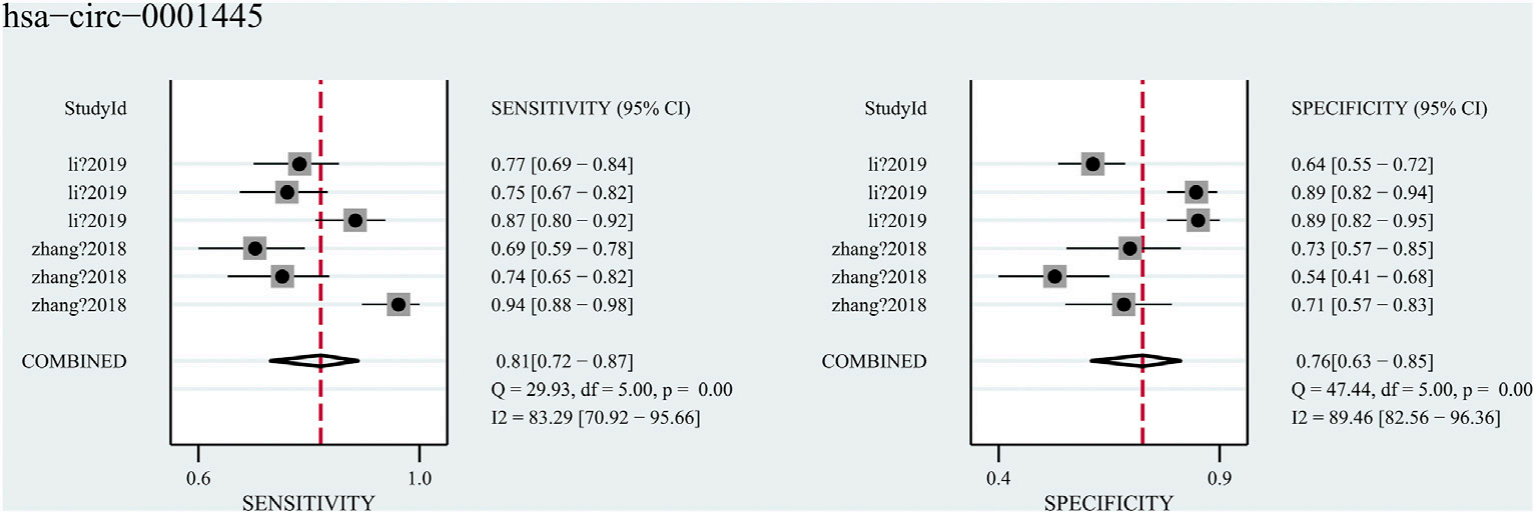
Forest plots for diagnostic accuracy of hsa_circ_0001445 in hepatocellular carcinoma (HCC).

Hsa_circ_0001445, was researched in two studies (Zhang et al. and Li et al.), which contained diagnose-related data and both focused on the diagnostic role of hsa_circ_0001445 in blood. A total of 755 blood samples (from 239 HCC patients, 200 cirrhosis patients, 161 hepatitis patients and 155 health subjects) were included in the two studies. The pooled sensitivity was 0.81 (95%CI: 0.72–0.87), and the pooled specificity was 0.76 (95%CI: 0.63–0.85) (Figure 6) with an AUC of 0.85 (95%CI: 0.82–0.88). However, significant heterogeneity (I^2^ = 88%, *p* < 0.001) existed in these data. Considering that the diagnostic accuracy of hsa_circ_0001445 might be various in different groups of people, we divided them into three subgroups-HCC vs cirrhosis, HCC vs hepatitis, and HCC vs health. The results of subgroup analysis were showed in Table 2. We found that the group HCC vs health has the highest sensitivity (0.90), specificity (0.82) and AUC (0.91), with no heterogeneity (I^2^ = 0, *p* = 0.554). Regrettably, the reliability of our results was limited by the sparse studies.

**TABLE 2:** The subgroup analysis of the diagnostic accuracy of hsa_circ_0001445.

Group	Sen	95%CI	Spe	95%CI	DOR	95%CI	AUC	95%CI	heterogeneity	
									I^2^	P
HCC vs hepatitis	0.75	0.69–0.80	0.76	0.45–0.92	9	1–60	0.80	0.76–0.84	88%	0.003
HCC vs cirrhosis	0.74	0.68–0.79	0.66	0.59–0.72	6	4–9	0.76	0.71–0.80	81%	0.023
HCC vs health	0.90	0.84–0.95	0.82	0.66–0.92	48	26–91	0.91	0.82–1.00	0	0.554
Overall	0.81	0.72–0.87	0.76	0.63–0.85	13	6–30	0.85	0.82–0.88	88%	< 0.000

Abbreviations: Sen: sensitivity; Spe: specificity; DOR: diagnostic OR; AUC: area under the curve; HCC: hepatocellular carcinoma.

##### 3.3.1.3 HCC Tissues vs Adjacent Tissues

Six studies explored the diagnostic role of circular RNAs from the comparisons between tissues adjacent to cancerous tissues and cancerous tissues. All seven studies were published before 2021. The pooled sensitivity was 0.78 (95% CI: 0.70–0.84). The pooled specificity was 0.78 (95%CI: 0.71–0.85). The pooled DOR, +LR, and −LR was 13 (95%CI: 7–25), 3.6 (95%CI: 2.5–5.2), and 0.28 (95%CI: 0.20–0.40), respectively. The Fagan’s nomogram plot analysis revealed values of 48% (positive) and 6% (negative) (Supplementary Figure S2C). The correlation coefficient of the threshold effect was 0.49 (*p* = 0.29): a threshold effect was absent. The pooled AUC was 0.85 (0.82–0.88). Cochran’s Q was 2.000 (*p* = 0.140) and I^2^ was 21%, which indicated low heterogeneity (Figure 4C).

##### 3.3.1.4 HCC Tissues vs Tissues From Patients With Cirrhosis or Hepatitis

Three studies explored the diagnostic accuracy of circular RNAs extracted from HCC tissues and the tissues of patients suffering from cirrhosis or hepatitis. All three studies were published before 2020. The pooled sensitivity was 0.56 (95%CI: 0.49–0.64). The pooled specificity was 0.76 (95%CI: 0.67–0.82). The pooled DOR, +LR, and −LR was 4 (95%CI: 3–6), 2.3 (95%CI: 1.8–3.0), and 0.58 (95%CI: 0.50–0.66), respectively. The Fagan’s nomogram plot analysis revealed values of 37% (positive) and 13% (negative) (Supplementary Figure S2D). The correlation coefficient of the threshold effect was −1.00 (*p* = 1): a threshold effect was absent. The pooled AUC was 0.70 (0.66–0.74). Cochran’s Q was 9.881 (*p* = 0.004) and I^2^ was 80%, which indicated moderate heterogeneity (Figure 4D).

#### 3.3.2 Function in HCC

After searching for target circular RNAs in related databases, we listed the circular RNAs related to the pathological mechanism of hepatocellular carcinoma, including hsa_circ_0001649, hsa_circ_0005075, CircZKSCAN1, hsa_circ_0001445, hsa_circ_0003998, Circ-104075, Circ-TCF4.85, Circ-CDYL, hsa_circ_0027345, hsa_circ_0051443, hsa_circ_0005397. Most of them worked as a miRNA sponge, except hsa_circ_0027345, forming a ternary complex with EZH2 and STAT3. Moreover, circ-104075, circ-TCF4.85, has-circ-0027345, and has-circ-5397 were only reported to be pathologically functional in HCC. Others were just focused on their clinic values. The results were showed in Supplementary Table S2. Their target genes consisted of SHPRH, DLC1, TIMP3, PCBP1, YAP-1, HDFG, HIF1AN, STAT3, RNF38, BAK1 and PDK2. Only hsa_circ_0001649 can activate its parental gene, SHPRH, to inhibit HCC progression *via* sponging miR-127–5p/miR-612/miR-4688. We also calculated the pooled sensitivity, specificity and AUC for two groups, function or not (Table 1). The pooled sensitivities were 0.82 (95%CI: 0.77–0.86) (function group) and 0.75 (95%CI: 0.67–0.82) (unknown function group), when the specificities were 0.80 (95%CI: 0.73–0.86) and 0.84 (95%CI: 0.80–0.87). As for AUC, they were 0.88 (95%CI: 0.84–0.90) (function group) and 0.87 (95%CI: 0.84–0.90) (unknown function group). As we knew, there was a tiny difference in AUC between two groups.

#### 3.3.3 Only in HCC or Not

We extracted several circular RNAs that only were reported to work in HCC, including hsa_circ_0003570, hsa_circ_0068669, hsa_circ_0128298, hsa_circ_000224, hsa_circ_00156, hsa_circ_000520, hsa_circ_0028502, hsa_circ_0076251, hsa_circ_0004001, hsa_circ_0004123, hsa_circ_0075792, hsa_circ_0009582, hsa_circ_0037120, hsa_circ_0140117, hsa_circ_0027345, hsa_circ_0051443, hsa_circ_0005397, hsa_circ_0006602, hsa_circ_0028861. Others were found that played some roles in other cancers, such as gastric cancer, colorectal cancer, glioma, bladder cancer and so on (Supplementary Table S2). In addition, circ-104075, circ-TCF4.85, hsa_circ_0027345, hsa_circ_0051443 and hsa_circ_0005397 could regulate specific target genes through sponging microRNAs or binding to proteins only in HCC. Similarly, the same statistical treatment happened in both of two groups-only in HCC or not. The pooled sensitivities were 0.79 (95%CI: 0.71–0.84) and 0.79 (95%CI: 0.73–0.83), equally in two groups, and the specificities were 0.84 (95%CI: 0.80–0.88) (only in HCC) and 0.78 (95%CI: 0.70–0.85) (not only in HCC). As for AUC, they were 0.89 (95%CI: 0.86–0.91) and 0.85 (95%CI: 0.82–0.88) (Table 1). Consequently, these circular RNAs only reported in HCC had a higher pooled specificity and an AUC.

#### 3.3.4 Quality Assessment and Publication Bias

**FIGURE 7:**
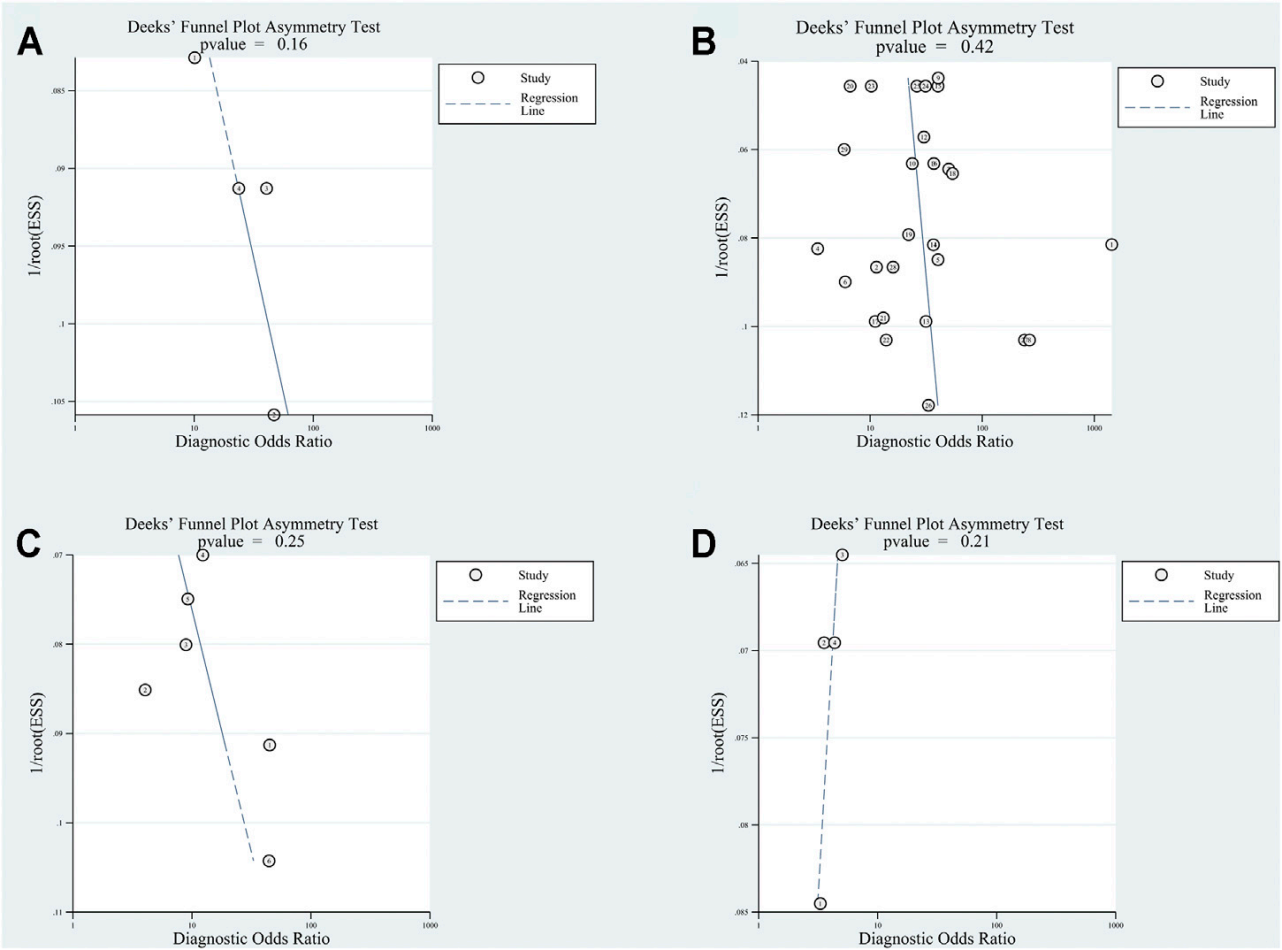
Deek’s Funnel test for circular RNAs in **(A)** exosomes; **(B)** serum/plasma; **(C)** HCC tissue vs adjacent tissue; **(D)** HCC tissues vs tissues from cirrhosis or chronic hepatitis cases.

The included articles were judged by QUADSA-2. The details were shown in Supplementary Figure S1. All articles were acceptable. Deeks’ funnel-plot asymmetry test was processed to ascertain if a publication bias was present in our analysis. The results, *p*-value of exosomes group, plasma/serum group, adjacent tissues group and tissues from patients with cirrhosis or hepatitis group were 0.16, 0.42, 0.25 and 0.21, demonstrating a low likelihood of the publication bias (Figure 7).

## 4 Discussion

Compared to radioactive or expensive imaging methods, biomarkers are easier to be evaluated because the measurements are objective and can be obtained in real time using a relatively innocuous and low-cost venipuncture procedure (Öberg et al., 2020). The potential biomarkers that could be used to detect HCC are AFP, glypican-3 (GPC3), des-gamma-carboxy prothrombin (DCP), miRNAs, and circular RNAs(Yu et al., 2020). The sensitivity and specificity of AFP for detecting HCC has been reported to be 39–64% and 76–91% (Oka et al., 1994; Okuda et al., 2000; Marrero and Lok, 2004), respectively. The sensitivity and specificity of the serum GPC3 level to detect HCC was reported to be 68 and 92%, but the results were influenced by the threshold effect and significant heterogeneity (Xu et al., 2019). According to a meta-analysis in 2014, the sensitivity and specificity of DCP to detect HCC was 71 and 84%, respectively (Zhu et al., 2014). However, those protein biomarkers relied on inconvenient enzyme-linked immunosorbent assays for rapid detection, taking serval hours.

We included 23 articles (5,135 persons) from 2016 to 2021 to explore the diagnostic role of circular RNAs in HCC detection. The pooled sensitivity and specificity were 0.80 and 0.83, respectively, with an AUC of 0.88. The result of Fagan test means circular RNAs could be used to diagnose HCC (when the pre-test probability is 20%), with 54% probability of correctly diagnosing HCC following a “positive” measurement and 6% probability of wrong diagnosing HCC following a “negative” result. These data suggested that circular RNAs were suitable biomarkers for HCC detection. An electrochemical detection assay of circular RNAs based on a combination of back-splice junctions and duplex-specific nucleases was first reported in 2019 (Jiao et al., 2020). Currently, application of an electrochemical POCT platform for DNA and glucose has been expanded to circular RNAs. This electrochemical POCT platform can be integrated readily with a smartphone using limited sample volumes to achieve a sensitive, accurate, real-time, and rapid analysis (Zhang et al., 2021).

Exosomes are nanoscale (30–150 nm) extracellular vesicles of endocytic origin shed by most types of cells. Exosomes circulate in interstitial fluid, blood, urine, saliva, and breast milk (Boriachek et al., 2018) and function in mediating intercellular communication, tumor microenvironment (Zhang and Wang, 2015), immune system (Yu et al., 2018), development and differentiation, cell signaling and viral replication (Alenquer and Amorim, 2015). In 2015, Li and coworkers firstly reported exosome-derived circular RNAs, and explored their potential diagnostic value (Li Y. et al., 2015). They identified 137,696 circular RNAs in the exosomes presenting in human serum, and >90% of the circular RNAs overlapped with known genes, including 82,892 multiexon circular RNAs, 27728 exon–intron circular RNAs, 6,709 intronic circular RNAs, 3,961 single-exon circular RNAs. Those data suggested that circular RNAs could be transferred actively from cells to exosomes, and indicated the underlying possibility of using circular RNAs as diagnostic markers for tumors (Wang et al., 2019).

Our meta-analysis showed that exosome-derived circular RNAs had a specificity of 0.91 and post-test probability of 66% (positive) and 8% (negative) as the low sensitivity (0.69) and AUC (0.82). Such relatively low sensitivity and AUC might result from included studies about hepatitis-related exosomes in blood, and a model using single circular RNA. Extracellular vesicle long RNAs (eight RNAs diagnostic model) have been reported to have an AUC of 0.98 with 89% sensitivity and 91% specificity for detecting HCC(Li Y. et al., 2019). According to a study focusing on miRNA, single miR-251–5p was 0.72 (AUC), when combination of three exo-miRNAs had a 0.95 AUC(Cho et al., 2020). Combination with traditional biomarkers was another way. As shown in Wang and others’ research, hsa_circ_0028861 plus AFP had an impressive development in sensitivity (0.67–0.76) (Wang et al., 2021).

Circular RNAs existing in blood or other bodily fluids are stable and can be used as biomarkers. Different from previous Jiang’s meta-analyses, in our meta-analysis, the serum/plasma group had a higher sensitivity (0.84 *vs*. 0.78) and specificity (0.83 *vs*. 0.78) compared with those from the adjacent-tissue group, and the AUC was similar (0.90 *vs*. 0.85). The diagnostic value from Fagan analysis similarly proved this (55–5% vs 48–6%). Hence, circular RNAs from serum/plasma or tissues were suitable diagnostic biomarkers for HCC. Besides, the relationship between expression in serum/plasma and that in HCC tissues from HCC patients required deeper exploration. The heterogeneity observed in the serum/plasma group might have been caused by diverse blood samples (healthy people, individuals suffering from cirrhosis or chronic hepatitis, or people not suffering from HCC) from our former meta-analysis (Nie et al., 2021). In addition, the heterogeneity also was caused by different publication years, single or multiple circular RNAs diagnostic models and sample size.

As for hsa_circ_0001445, a circular RNA with a sensitivity (0.81), specificity (0.76) and an AUC (0.85) in combining two studies, might be a suitable diagnostic blood biomarker for HCC. Following development of liver cirrhosis in patients with chronic hepatitis, liver disease may continue to progress and evolve into hepatocellular carcinoma. Some circular RNAs have been proved to participate in this process. Circular RNA cMTO1 was reported to suppress liver fibrosis and hepatocellular carcinoma progression by acting as a sponge of microRNA (Han et al., 2017; Jin et al., 2020). This might explain the worse diagnostic efficiency of circular RNA happening in hepatitis or cirrhosis patients and HCC patients. Otherwise, the abundance of hsa_circ_0001445 changed gradually in HCC, cirrhosis, hepatitis patients and healthy subjects in both of studies. We could conjecture that hsa_circ_0001445 functioned in the process of hepatitis-cirrhosis-hepatocellular carcinoma, which required deeper researches to prove.

Multi-circular RNAs models had a higher diagnostic accuracy. To improve the accuracy of detection of HCC using serum/plasma circular RNAs, a preferable strategy may be to combine several circular RNAs with conventional biomarkers. For example, in a large-scale multicenter study, the AUC of hsa_circ_0000976 was 0.70. Upon combining hsa_circ_0000976 with two other circular RNAs (hsa_circ_0007750 and hsa_circ_0139,897), the AUC became 0.86. With a combination of those three circular RNAs and AFP, the AUC reached 0.87 (Sun X.-H. et al., 2020). Besides serum/plasma circular RNAs, circ_0000798 extracted from peripheral blood mononuclear cells had an AUC of 0.70 for distinguishing HCC patients from healthy controls (Lei et al., 2019). Wang and collaborators reported that circSATD3 in peripheral venous blood could predict microvascular invasion with an AUC of 0.637 (Wang et al., 2020). Moreover, in a meta-analysis about the diagnostic accuracy of combination of circular RNAs and AFP in detecting HCC, their combination showed a higher clinic application value (Nie et al., 2021). Thus, the diagnostic value of circular RNAs in different components of blood merits further investigation.

In the subgroups of cirrhosis and hepatitis, sensitivity (0.56) and specificity (0.76) were lowest in all groups. The post-test possibility was in the same condition. This result was similar to our previous study on serum/plasma circular RNAs, and indicates the limitation of using circular RNAs to distinguish HCC patients from patients suffering from cirrhosis or chronic hepatitis. Therefore, more studies are required to ascertain the more effective circular RNAs for detection of cirrhosis and chronic hepatitis.

Circular RNAs from exosomes or serum/plasma shared more clinical diagnostic value than circular RNAs from tissues. Circular RNAs are ideal candidates as biomarkers in “liquid biopsies” because they are expressed in tissue- and developmental stage-specific manners, and are also found stable existence in bodily fluids (Arnaiz et al., 2019). Liquid biopsy is a non-invasive method that uses body fluids (e.g., blood, plasma, serum, urine, gastric juice) to reflect the disease state (Reimers and Pantel, 2019). This method could be used to detect HCC at an early stage before insensitive and expensive imaging examinations. Seventy percent of HCC patients detected at an early stage can achieve 5-years survival with suitable therapies (Padhya et al., 2013). In addition, the detection of circular RNAs in blood or in exosomes has been widely explored. A virus-mimicking vesicle (Vir-FV) method was reported that enabled rapid, efficient, and high-throughput detection of exosome-derived miRNAs within 2 h (Gao et al., 2019). Getulio et al. detected extracellular vesicle RNAs using molecular beacons, which was fast, simple and specific (Oliveira et al., 2020). Thus, self-testing has become available with POCT platforms and similar technologies.

The group function and group non-function seemed no difference in diagnostic value. Maybe this result could be explained by that some non-function circular RNAs just were not found out their mechanism in HCC at present. Obviously, most studies paid their close attention to the role of circular RNAs in the growth and metastasis of HCC. Just like included studies in our meta-analysis, most of circular RNAs were found to regulate the growth and metastasis, except hsa_circ_0003998, which could promote epithelial to mesenchymal transition, and circ-CDYL, which could cause stem-like characteristics. Several researchers have explored how circular RNAs influenced resistance. For example, circ-SORE and circ-FN1 were reported to induce HCC sorafenib resistance (Xu et al., 2020; Yang et al., 2020). However, regulation mechanisms of circular RNAs in immunity escape, metabolism, cancer stem cell or other areas were lacking.

Analysis of circular RNAs reported only in HCC or not, we found that these circular RNAs only reported in HCC had a higher pooled specificity and AUC, which meant they might have a more ideal diagnostic accuracy. Obviously, the characteristics of circular RNAs still stayed unknown. The same circular RNA possessed varied regulation mechanisms in different cancers, known or unknown. Hsa_circ_0001445 could regulate VEGFA mRNA through binding to SRSF1 in glioblastoma, while in HCC, it sponged miR-17–3p and miR-181b-5p to induce the expression of TIMP3 in order to inhibit growth and metastasis. Not only in cancers, it also inhibited ox-LDL-induced HUVECs inflammation by regulating miR-640 (Cai et al., 2020). In a word, the mechanisms of circular RNAs were complex and unexplainable. Thus, this area needed more attention and researches.

Our meta-analysis had four main limitations. First, most studies were carried out in China, which limited the generalizability of our findings. This phenomenon is due (at least in part) to the high incidence of HCC-related deaths in China. Second, the comparatively poor performance of circular RNAs for identifying patients with HCC from patients with cirrhosis or hepatitis weakened the goal of HCC surveillance (which is to diagnose and treat HCC early in high-risk individuals to improve long-term outcomes) (Yang and Kim, 2012). Hence, more suitable circular RNAs or models must be developed for screening, especially in high-risk individuals. The American Association for the Study of Liver Diseases and Asian Pacific Association for the Study of the Liver define “high-risk individuals” as Asian men (age >40 years ears) or Asian women (age >50 years), individuals with a family history of HCC, and African or African–American individuals, all with hepatitis-B-virus infection or individuals with cirrhosis (Omata et al., 2017; Marrero et al., 2018). Third, the level of evidence was low as a result of the lack of randomized controlled trials or prospective studies. Fourth, high heterogeneity remained in some subgroups (e.g., circular RNAs from serum/plasma).

## 5 Conclusion

Our meta-analysis suggested the marked diagnostic accuracy of circular RNAs for detecting HCC. The diagnostic accuracy varied among subgroups. The circular RNAs in exosomes and serum/plasma were suitable for liquid biopsies. In addition, more high-quality, multiple-central and multiple-circular-RNAs studies were needed to explore the value of circular RNAs for detecting HCC.

## Data Availability

The original contributions presented in the study are included in the article/Supplementary Material, further inquiries can be directed to the corresponding author.
