# Supplementary material for: Diagnostic Accuracy of Circular RNAs in Different Types of Samples for Detecting Hepatocellular Carcinoma: A Meta-Analysis
Source: Front Genet. 2021 Dec 21;12:794105. doi: 10.3389/fgene.2021.794105 (PMC8724259; doi:10.3389/fgene.2021.794105)

| **Table S1. List of studies about circRNAs included in the meta-analysis** | | | | | | | | | | | | | | |
| --- | --- | --- | --- | --- | --- | --- | --- | --- | --- | --- | --- | --- | --- | --- |
| **Study** | **Year** | **country** | **biomarker** | **detection** | **Study type** | **Control type** | **Cut-off value** | **TP** | **FP** | **FN** | **TN** | **Sen** | **Spe** |  |
| Qin et al. | 2016 | China | hsa_circ_0001649 | qPCR | Case control study | Adjacent tissue | 7.85×10^-4^ | 72 | 28 | 17 | 61 | 0.810 | 0.690 |  |
| Shang et al. | 2016 | China | hsa_circ_0005075 | qPCR | Case control study | Adjacent tissue | N/A | 50 | 6 | 10 | 54 | 0.833 | 0.900 |  |
| Fu et al. | 2018 | China | hsa_circ_0003570 | qPCR | Case control study | Cirrhosis tissue | 12.24 | 48 | 19 | 59 | 118 | 0.449 | 0.863 |  |
| Yao et al. | 2018 | China | hsa_circ_0068669 | qPCR | Case control study | hepatitis tissue | N/A | 41 | 21 | 29 | 49 | 0.590 | 0.710 |  |
| Yao et al. | 2018 | China | CircZKSCAN1 | qPCR | Case control study | Adjacent tissue | N/A | 84 | 28 | 18 | 74 | 0.822 | 0.724 |  |
| Chen et al. | 2018 | China | hsa_circ_0128298 | qPCR | Case control study | Adjacent tissue | N/A | 53 | 15 | 25 | 63 | 0.674 | 0.805 |  |
| Matboli et al. | 2018 | Egypt | hsa_circ_000224 | qPCR | Case control study | serum | N/A | 65 | 3 | 3 | 33 | 0.956 | 0.927 |  |
|  | 2018 | Egypt | hsa_circ_00156 | qPCR | Case control study | serum | N/A | 50 | 6 | 18 | 30 | 0.735 | 0.823 |  |
|  | 2018 | Egypt | hsa_circ_000520 | qPCR | Case control study | serum | N/A | 66 | 4 | 2 | 32 | 0.971 | 0.896 |  |
| Zhang et al. | 2018 | China | hsa_circ_0001455 | qPCR | Case control study | plasma | N/A | 98 | 15 | 6 | 37 | 0.942 | 0.712 |  |
|  | 2018 | China | hsa_circ_0001455 | qPCR | Case control study | plasma | N/A | 77 | 26 | 27 | 31 | 0.740 | 0.544 |  |
|  | 2018 | China | hsa_circ_0001455 | qPCR | Case control study | plasma | N/A | 72 | 12 | 32 | 32 | 0.692 | 0.727 |  |
| Jiang et al. | 2019 | China | hsa_circ_0028502 | qPCR | Case control study | Cirrhosis and hepatitis tissue | 9.595 | 58 | 30 | 42 | 77 | 0.580 | 0.721 |  |
|  | 2019 | China | hsa_circ_0076251 | qPCR | Case control study | Cirrhosis and hepatitis tissue | 12.340 | 64 | 31 | 36 | 76 | 0.640 | 0.713 |  |
| Li et al. | 2019 | China | CircSMARCA5 | qPCR | Case control study | plasma | 15.28×10^-2^ | 117 | 11 | 18 | 92 | 0.867 | 0.893 |  |
|  | 2019 | China | CircSMARCA5 | qPCR | Case control study | plasma | 9.385×10^-2^ | 101 | 13 | 34 | 104 | 0.748 | 0.889 |  |
|  | 2019 | China | CircSMARCA5 | qPCR | Case control study | plasma | 10.34×10^-2^ | 104 | 52 | 31 | 91 | 0.770 | 0.637 |  |
| Qiao et al. | 2019 | China | hsa_circ_0003998 | qPCR | Case control study | plasma | N/A | 84 | 20 | 16 | 80 | 0.840 | 0.800 |  |
|  | 2019 | China | hsa_circ_0003998 | qPCR | Case control study | plasma | N/A | 80 | 8 | 20 | 42 | 0.800 | 0.840 |  |
| Zhang et al. | 2019 | China | Circ-104075 | qPCR | Case control study | plasma | N/A | 97 | 1 | 4 | 59 | 0.960 | 0.983 |  |
| Gao et al. | 2020 | China | Circ-TCF4.85 | qPCR | Case control study | Adjacent tissue | N/A | 40 | 6 | 6 | 40 | 0.868 | 0.870 |  |
| Wei et al. | 2020 | China | Circ-CDYL | qPCR | Case control study | Adjacent tissue | N/A | 43 | 20 | 26 | 49 | 0.630 | 0.710 |  |
| Sun et al. | 2020 | China | hsa_circ_0004001 | qPCR | Case control study | serum | 51.43 | 54 | 8 | 17 | 33 | 0.762 | 0.813 |  |
|  | 2020 | China | hsa_circ_0004123 | qPCR | Case control study | serum | 221.7 | 47 | 6 | 24 | 34 | 0.667 | 0.844 |  |
|  | 2020 | China | hsa_circ_0075792 | qPCR | Case control study | serum | 79.48 | 64 | 9 | 7 | 31 | 0.905 | 0.781 |  |
| Wu et al. | 2020 | China | hsa_circ_0009582 | qPCR | Retrospective | plasma | N/A | 121 | 60 | 59 | 300 | 0.673 | 0.833 |  |
|  | 2020 | China | hsa_circ_0037120 | qPCR | Retrospective | plasma | N/A | 155 | 69 | 25 | 291 | 0.861 | 0.807 |  |
|  | 2020 | China | hsa_circ_0140117 | qPCR | Retrospective | plasma | N/A | 148 | 37 | 32 | 323 | 0.822 | 0.897 |  |
|  | 2020 | China | combination | qPCR | Retrospective | plasma | N/A | 147 | 45 | 33 | 315 | 0.817 | 0.875 |  |
| Yu et al. | 2020 | China | circPanel | qPCR | Case control study | plasma | N/A | 248 | 30 | 42 | 206 | 0.855 | 0.873 |  |
|  | 2020 | China | circPanel | qPCR | Case control study | plasma | N/A | 248 | 8 | 42 | 68 | 0.855 | 0.895 |  |
|  | 2020 | China | circPanel | qPCR | Case control study | plasma | N/A | 248 | 11 | 42 | 69 | 0.855 | 0.863 |  |
|  | 2020 | China | circPanel | qPCR | Case control study | plasma | N/A | 248 | 11 | 42 | 69 | 0.855 | 0.863 |  |
|  | 2020 | China | circPanel | qPCR | Case control study | plasma | N/A | 133 | 29 | 19 | 125 | 0.875 | 0.812 |  |
|  | 2020 | China | circPanel | qPCR | Case control study | plasma | N/A | 133 | 8 | 19 | 42 | 0.875 | 0.840 |  |
|  | 2020 | China | circPanel | qPCR | Case control study | plasma | N/A | 133 | 13 | 19 | 41 | 0.875 | 0.759 |  |
|  | 2020 | China | circPanel | qPCR | Case control study | plasma | N/A | 133 | 8 | 19 | 42 | 0.875 | 0.840 |  |
| Sun et al. | 2020 | China | circ-LRIG3 | qPCR | Case control study | plasma | N/A | 29 | 4 | 7 | 32 | 0.792 | 0.892 |  |
| Chen et al. | 2020 | China | hsa_circ_0051443 | qPCR | Case control study | exosome | N/A | 41 | 5 | 19 | 55 | 0.682 | 0.914 |  |
| Zhu et al. | 2020 | China | hsa_circ_0004277 | qPCR | Case control study | exosome | N/A | 35 | 2 | 25 | 58 | 0.583 | 0.967 |  |
| Liu et al. | 2021 | China | hsa_circ_0005397 | qPCR | Case control study | plasma | 0.914 | 148 | 148 | 32 | 212 | 0.820 | 0.588 |  |
| Guo et al. | 2021 | China | hsa_circ_0006602 | qPCR | Case control study | exosome | N/A | 67 | 2 | 20 | 28 | 0.770 | 0.930 |  |
| Wang et al. | 2021 | China | hsa_circ_0028861 | qPCR | Case control study | exosome | N/A | 38 | 18 | 18 | 86 | 0.680 | 0.830 |  |
| Abbreviations: Sen: sensitivity; Spe: specificity; TP: true-positives; TN: true-negatives; FP: false-positives; FN: false-negatives; AUC: area under the curve; qPCR: quantitative polymerase chain reaction. | | | | | | | | | | | | | | |

| **Table S2. Overview of the identified hepatocellular carcinoma-associated circular RNAs** | | | |
| --- | --- | --- | --- |
| **Circular RNA** | **Type of disease** | **Function in HCC** | **Target gene** |
| hsa_circ_0001649 | gastric carcinoma; colorectal cancer; endometriosis; hepatocellular carcinoma; osteosarcoma; cholangiocarcinoma; glioma; pancreatic ductal adenocarcinoma; retinoblastoma; | sponging miR-127-5p/miR-612/miR-4688 | SHPRH (parental gene) |
| hsa_circ_0005075 | Colorectal cancer; hepatocellular carcinoma; | sponging miR-431 |  |
| hsa_circ_0003570 | hepatocellular carcinoma |  |  |
| hsa_circ_0068669 | hepatocellular carcinoma |  |  |
| CircZKSCAN1 | hepatocellular carcinoma; bladder cancer; non-small lung cancer | sponging miR-873-5p | DLC1 |
| hsa_circ_0128298 | hepatocellular carcinoma |  |  |
| hsa_circ_000224 | hepatocellular carcinoma |  |  |
| hsa_circ_00156 | hepatocellular carcinoma |  |  |
| hsa_circ_000520 | hepatocellular carcinoma |  |  |
| hsa_circ_0001445 | coronary artery disease; osteoporosis; hepatocellular carcinoma; glioblastoma multiforme; cervical cancer | sponging miR-17-3p/miR-181b-5p | TIMP3 |
| hsa_circ_0028502 | hepatocellular carcinoma |  |  |
| hsa_circ_0076251 | hepatocellular carcinoma |  |  |
| hsa_circ_0003998 | hepatocellular carcinoma; lung adenocarcinoma; non-small cell lung cancer | sponging miR-143-3p | PCBP1 |
| Circ-104075 | hepatocellular carcinoma | sponging miR-582-3p | YAP-1 |
| Circ-TCF4.85 | hepatocellular carcinoma | sponging miR-486-5p |  |
| Circ-CDYL | hepatocellular carcinoma; multiple myeloma; mantle cell lymphoma; | sponging miR-892a/miR-328-3p | HDGF/HIF1AN |
| hsa_circ_0004001 | hepatocellular carcinoma |  |  |
| hsa_circ_0004123 | hepatocellular carcinoma |  |  |
| hsa_circ_0075792 | hepatocellular carcinoma |  |  |
| hsa_circ_0009582 | hepatocellular carcinoma |  |  |
| hsa_circ_0037120 | hepatocellular carcinoma |  |  |
| hsa_circ_0140117 | hepatocellular carcinoma |  |  |
| hsa_circ_0027345 | hepatocellular carcinoma | binding to EZH2 | STAT3 |
|  |  | sponging miR-449a | RNF38 |
| hsa_circ_0051443 | hepatocellular carcinoma | sponging miR-331-3p | BAK1 |
| hsa_circ_0004277 | colorectal cancer; acute myeloid leukemia; hepatocellular carcinoma |  |  |
| hsa_circ_0005397 | hepatocellular carcinoma | sponging miR-326 | PDK2 |
| hsa_circ_0006602 | hepatocellular carcinoma |  |  |
| hsa_circ_0028861 | hepatocellular carcinoma |  |  |


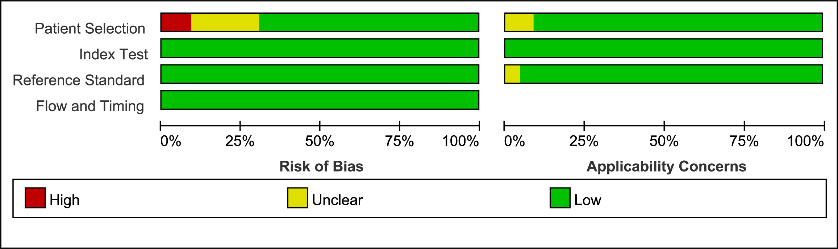

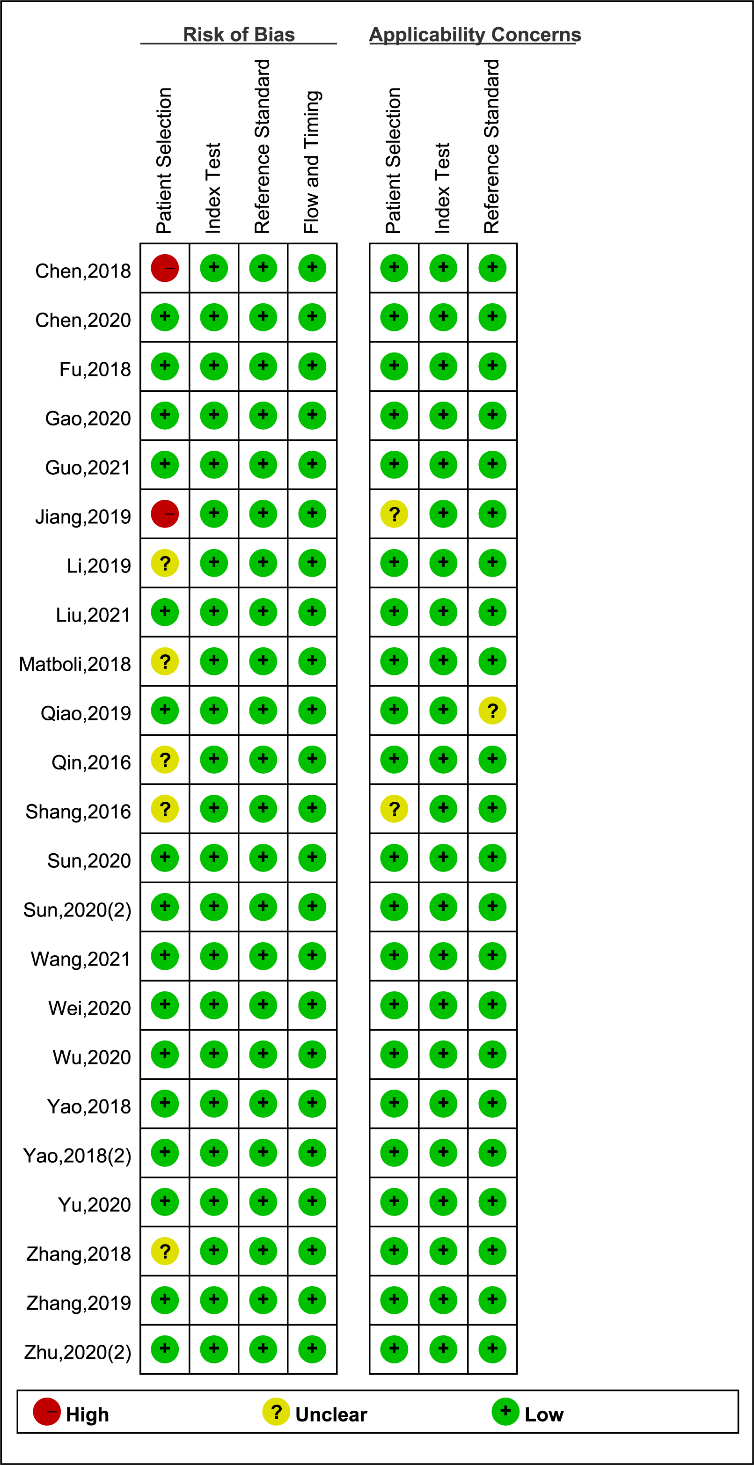
**Figure S1. Methodological quality of the included studies (QUADAS-2 results).**

**Figure S2. Fagan plot analysis for: (A) circRNAs in exosomes; (B) circRNAs in serum/plasma; (C) circRNAs in HCC tissue vs. adjacent tissue; (D) circRNAs in HCC tissues vs. tissues from cirrhosis or chronic hepatitis cases**


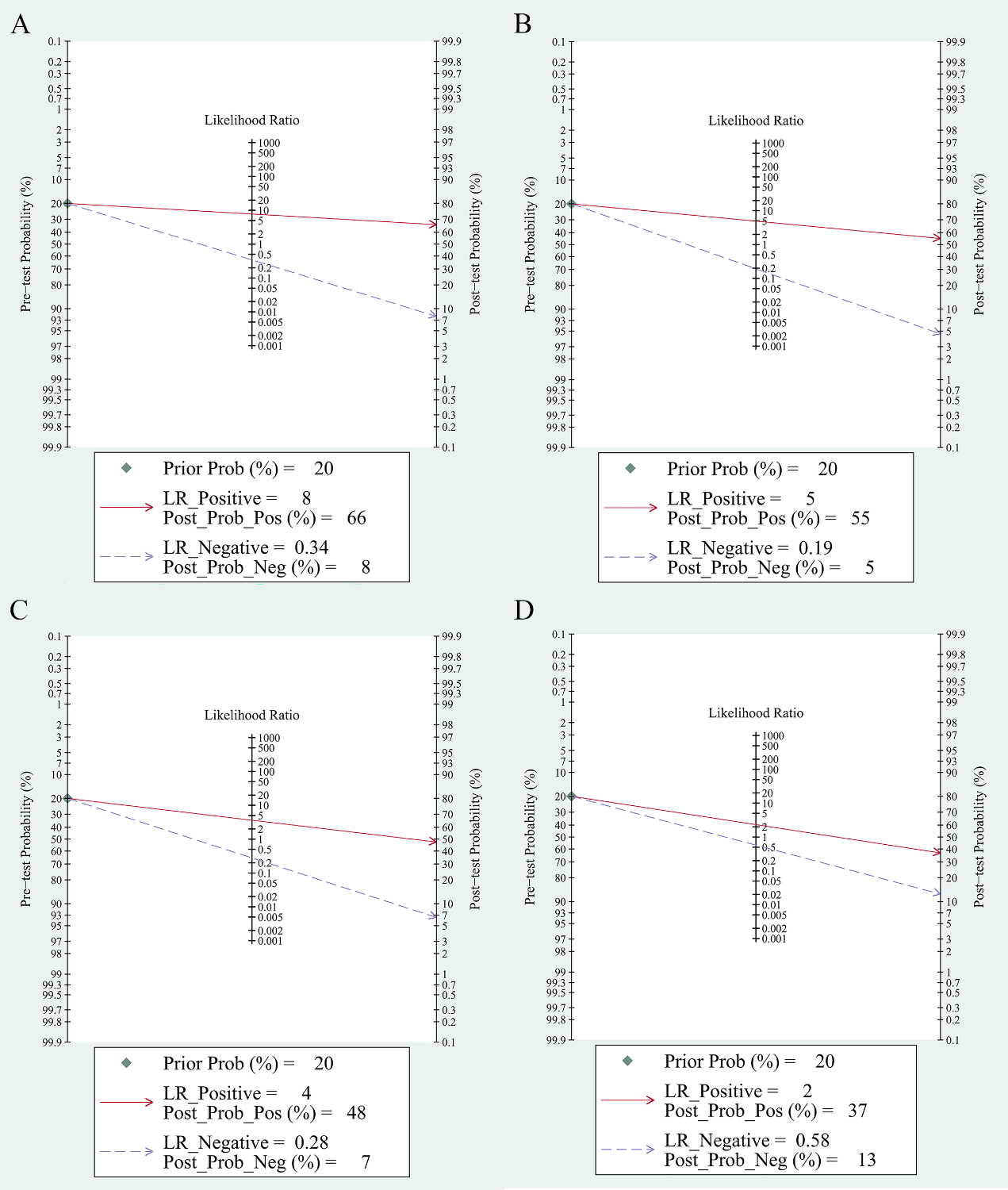

Supplement: Supplementary file 1 [file DataSheet1.docx]
